# Molecular Approaches to Safe and Controlled Engineered T-cell Therapy

**Published:** 2018

**Authors:** R. S. Kalinin, A. V. Petukhov, V. D. Knorre, M. A. Maschan, A. V. Stepanov, A.G. Gabibov

**Affiliations:** M.M. Shemyakin and Yu.A. Ovchinnikov Institute of Bioorganic Chemistry, Russian Academy of Sciences, Miklukho-Maklaya Str. 16 /10, Moscow, 117997, Russia; Dmitrii Rogachev Federal Research Center for Pediatric Hematology, Oncology and Immunology, Samory Mashela Str. 1, Moscow, 117997, Russia

**Keywords:** chimeric antigen receptors, T-cells, cell therapy, cancer cells

## Abstract

Chimeric antigen receptor-modified T-cell therapy (CAR-T therapy) is one of the fastest developing areas of immuno-oncology. Over the past decade, it has revolutionized the cell therapy modality and expedited its pace of development, from optimization of the structure of chimeric antigen receptors and animal model experiments to successful clinical application. The initial designs of the CAR configuration focused on increasing T-cell activation, cytotoxicity, and persistence. However, the first attempts to treat patients with CAR T cells have demonstrated the need for increased safety and controlled activation of genetically modified T cells. Herein, we summarize the different molecular approaches to engineering chimeric antigen receptors for reducing the potential clinical risks of T-cell therapy.

## INTRODUCTION

Adoptive cell immunotherapy was first used to treat metastatic sarcoma in 1985 and remains one of the most promising trends in cancer treatment [1, 2]. In this therapy, autologous T cells are isolated, activated, expanded, and infused back into the patient, resulting in partial regression or eradication of the tumor [3-6]. Application of autologous T cells prevents the development of the graft-versus-host disease (GVHD) and, importantly, enhances the persistence of therapeutically active cells [7-9]. However, adoptive immunotherapy shows lack of effectiveness in most cases [10]. The next step in the evolution of this therapy was to engineer T cells that could specifically recognize tumor cells and circumvent their immunosuppressive mechanisms. One of the ways to modify T cells is to insert an artificial T-cell receptor (TCR) targeting tumor-associated antigens (TAAs) [11]. Unfortunately, TCR-modified T cells can recognize only the protoasome-processed antigens presented by major histocompatibility complex class I (MHC I). The most recent approach, which consists in modifying T cells with chimeric antigen receptor (CAR) genes, is devoid of these shortcomings: this method helps T cells recognize the native antigens presented on the cancer cell membrane irrespective of MHC I. In terms of its structure, a CAR consists of three functional components: the extracellular antigen recognition domain; the transmembrane domain; and the intracellular component that comprises the T-cell activation domain of CD3ζ and, depending on what “generation” a receptor belongs to, different costimulatory domains (*Figure A*) [12]. Z. Eshhar and colleagues (the Weizmann Institute of Science, Israel) were the first to report on the use of a technique employing MHC I-independent recombinant antigen receptors back in the late 1980s [13]. This approach eventually evolved into the CAR T-cell therapy and yielded promising results in studies focused on hematological malignancies. Thus, clinical trials of CAR-modified T cells (CAR T cells) targeting the B-lymphocyte antigen CD19 have demonstrated that they are efficacious in the treatment of chemotherapy-resistant tumors of B-cell origin [14-18]. Finally, the Food and Drug Administration (FDA) in 2017 approved CAR T-cell products (Kymriah manufactured by Novartis and Yescarta manufactured by Kite Pharma) targeting CD19 for the treatment of acute lymphoblastic leukemia (ALL).

## THE RISKS ASSOCIATED WITH CAR T-CELL THERAPY

The earliest clinical trials of CAR-T therapy demonstrated its exceptional efficacy. Infusion of modified T cells resulted in an exponential increase in the T-cell count and active elimination of tumor cells already after the first several weeks [19]. The dark side of such an efficacious therapy is the high risk of developing systemic and life-threatening adverse events, primarily hypercytokinemia (cytokine storm, cytokine cascade, and cytokine release syndrome) or the tumor lysis syndrome [20-23]. These complications may trigger the multiple organ dysfunction syndrome and eventually cause death. These T cell-induced complications can be eliminated using cytostatic and cytotoxic corticosteroids [24]; however, these medications suppress all T cells and cause a number of side effects, such as systemic organ failure [25]. Another problem related to the application of CAR T cells consists in their nonspecific cytotoxicity; this issue becomes especially topical in the treatment of solid tumors as it is arduous to choose specific TAAs for this type of tumors [26-29]. Thus, clinical trials aimed at evaluating CAR T cells targeting carbonic anhydrase IX, which is hyperexpressed in renal cell carcinoma cells but is also present in normal tissues, including liver, have revealed that CAR T cells exhibit the nonspecific cytotoxicity that causes complications in patients [26, 28]. Furthermore, the use of HER2-specific CAR for a patient with metastatic colon cancer results in a rapid and intense cross reaction to healthy lung cells expressing HER2 at low levels and patient death immediately after the infusion of CAR T cells [30]. The methods for controlling the expansion and cytotoxicity of T cells already infused into a patient need further elaboration in order to improve safety and eliminate the current drawbacks, such as delayed cross-reactivity and toxicity after a successful CAR T-cell therapy [6, 31]. Herein, we summarize the different molecular approaches to safe and controlled T-cell therapy.

## APPLICATION OF THE HERPES SIMPLEX VIRUS THYMIDINE KINASE (HSV-TK) GENE

Herpes simplex virus thymidine kinase has long been used in both laboratory and clinical studies to induce cell death. HSV-TK phosphorylates ganciclovir to ganciclovir monophosphate, which is further stepwise converted to di- and triphosphates by cellular kinases (*Figure B*) [32-34]. Ganciclovir triphosphate is incorporated into DNA during the elongation and replication stages, thus disrupting the DNA polymerase function and causing cell death [35, 36]. Ganciclovir phosphorylated by viral thymidine kinase causes ligand-independent CD95 aggregation, which induces the formation of a Fas-associated protein with a death domain (FADD) and activates caspase-8 [37]. Elimination of the modified cells using ganciclovir and cells carrying the HSV thymidine kinase gene is the best studied technique with verified safety and efficacy [34, 38]. However, this approach also has some drawbacks consisting in the immunogenicity of HSV-TK [39]. Clinical trials have revealed that T-cell elimination is not a fast process as it requires DNA replication for the nucleotide analogue to be incorporated into the genome [38, 40]. Furthermore, this therapy cannot be performed if a patient has a herpes infection. Despite the apparent limitations of the approach, neither acute toxicity nor an immunogenic response to HSV-TK has been observed in clinical trials evaluating allogeneic HSV-TK-transduced T cells [41]. In two patients, ganciclovir was used to treat GVHD and complete elimination of HSV-TK+ was achieved; however, GVHD was successfully mitigated in only one patient. No immune response to HSV-TK was observed in the clinical trial [42], but GVHD did not occur in this study (possibly, because of the immunocompromised status of the patients and the low dose of infused T cells).

## APPLICATION OF CHEMICALLY INDUCIBLE CASPASE-9

The use of chimeric molecules based on pro-apoptotic signaling proteins that are capable of dimerization and activation in the presence of low-molecular-weight compounds is an interesting and promising approach to a controlled induction of apoptosis in CAR T cells [43, 44]. One of the most vivid examples is chimeric caspase-9 (iCasp9) [45], which consists of two key components: truncated caspase-9 and a fragment of the FKPB12-binding protein carrying a F36V mutation (FK506). This chimeric protein is dimerized in the presence of rimiducid (AP1903), thus inducing the apoptotic cascade (*Figure B)*. The iCasp9 system is apparently advantageous over HSV-TK. First, it consists of human gene products exhibiting low potential immunogenicity. Second, administration of the medicinal product does not produce significant adverse effects and results in selective elimination of CAR T cells only [46]. In addition, iCasp9 remains functionally active even in T cells that exhibit enhanced expression of anti-apoptotic proteins [43, 47-49]. The key advantage of iCasp9 over HSV-TK is that the former system acts very rapidly. Exposure to AP1903 for several hours leads to the elimination of CAR T cells. The efficacy of iCasp9 was proved for CAR T cells with different targets (CD19, CD20, and CD30). Clinical trials involving patients with lymphoma (NCT02274584) have also demonstrated that this approach is safe and efficacious [50].

## ELIMINATION OF CAR T CELLS BY MONOCLONAL ANTIBODIES

In the past decade, monoclonal antibodies (mAbs) have been routinely used in cancer therapy. Novel chimeric antigen receptors have been designed using therapeutic antibody variable domains. Interestingly, some antibodies that have already passed all the required clinical trials and have been approved by the FDA can be used for eliminating CAR T cells if patients develop complications from cellular therapy [51-53]. In order to eliminate T cells by mAbs, a proper antigen needs to appear on the surface of CAR T cells (Figure C). The same antigen can be employed to select CART+ cells following the modification of T cells [9]. The pioneering studies in this area were the experiments on the transduction of T cells with a CD20 molecule and infusion of anti-CD20 monoclonal antibodies, which proved themselves to be effective in the therapy of lymphoproliferative disorders of B-cell nature [54-56]. A similar system has been designed for the truncated form of the epidermal growth factor receptor (tEGFR) and acts as a target for the currently marketed medicinal product cetuximab [52]. tEGFR has undergone several clinical trials; however, application of cetuximab has not been found justifiable enough. In some studies, the mAb epitope was integrated into the sequence of the extracellular domain of the CAR. This approach was employed in a preclinical study where a 10-aa tag of c-myc was inserted into the recombinant TCR sequence [9, 51]. However, when considering mAbs for clinical application, one should take into account the intrinsic cytotoxicity of the antibody and the possible complications [9].

## SELF-/NONSELF DISCRIMINATION

Researchers have for a long time faced the problem of the choice of a TAA that would target tumor cells only, since it is extremely difficult to select unique antigens for most types of cancer cells. However, it is possible to select deterministic antigen patterns that are typical both of healthy and tumor cells. Fedorov et al. [57] suggested using an additional inhibitory chimeric receptor (iCAR) that protects normal cells against the nonspecific cytotoxicity of CAR T cells: when interacting with the antigens of healthy cells, it transmits an inhibitory signal (*Figure D*). The iCAR-modified cells inhibit the signals from the main CAR through the extracellular domain of PD-1 or CTLA-4. The key advantage of this approach is that the inhibitory effect is reversible and the T cells can still function when they subsequently encounter a tumor cell [57]. Such factors as proper selection of the expression level of the chimeric receptor, the balance between the affinities of the recognition domains, variability of the set of antigens presented on cancer and healthy cells, as well as the individual characteristics of each patient, significantly limit the clinical application of iCARs [57].

## ELIMINATION OF A CELL CARRYING A CERTAIN COMBINATION OF ANTIGENS

The problem of searching for tumor-specific antigens is especially relevant for solid tumors [58]. Therefore, it has been suggested that the selectivity and safety of CAR T cells can be enhanced if two receptors targeting different tumor antigens are expressed. It is not until all the CARs (one receptor may contain the CD3ζ stimulatory domain, while the other may carry CD28) have recognized their targets that a T cell receives stimulation sufficient for its activation (*Figure F*) [59-63]. This dual targeting system allows one to significantly reduce the intensity of adverse effects even in the absence of a specific tumor antigen [62]. W. Wilkie et al. compared CAR-modified T cells carrying two receptors and control CAR-modified T cells having one receptor with all its intracellular domains and found that despite the identical efficacy *in vivo*, the level of interleukin-2 secretion was significantly lower in the T cells with two receptors [63]. However, when using dual-targeted CARs, one should take into account that the efficiency of cell elimination and proliferation will directly depend on the balance between the signals from two receptors, with the optimal balance lying in a rather narrow range. A strong difference in the quantities of the two target antigens presented on tumor cells or the absence of one antigen may render cellular therapy ineffective.

In another strategy, a synthetic Notch receptor (synNotch) was designed: this receptor binds to the second antigen on a tumor cell and triggers the expression of CAR inside the T cells via transcription factors (Figure E) [64]. In its turn, CAR binds to its antigen presented on the tumor cell and activates the cytotoxicity of this CAR-modified T cell. Localized suppression of tumor cells is achieved thanks to this mechanism, without the risk of exhibiting nonspecific cytotoxicity with respect to healthy tissues.

Hence, using two different antigens present on tumor cells for recognition broadens the possible range of target antigens for CAR T cells and simultaneously reduces the toxicity that would be observed for conventional CAR T cells. However, neither this method nor modification of iCARs allows real-time control over CAR T cells and the intensity of their activity [65]. The constantly updated human protein reference databases are another solution to the problem of searching for an antigen that targets healthy cells [66]. MHC can also be a promising antigen that discriminates between healthy cells and tumor ones: it is expressed on the surface of almost all healthy cells, while MHC expression in cancer cells is downregulated to suppress the immune response [67].

## CONTROLLING THE EXPRESSION OF THE CHIMERIC ANTIGEN RECEPTOR GENE

Since activation and the cytotoxicity of modified T cells directly depend on the quantity of the receptor presented on the cell membrane, the effectiveness of cell therapy can be controlled by regulating the expression of the chimeric antigen receptor gene. Inducible promoters have been used to regulate gene expression over the past decades. The tetracycline-responsive promoter system is a convenient tool for regulating gene expression in eukaryotic cells. CAR expression in modified T cells can be regulated through dosed insertion of a regulatory molecule. In one case, doxycycline inhibited CAR expression [68]. Contrariwise, CAR was expressed only in the presence of doxycycline in another case [69]. The convenience of this method is that it allows one to regulate cytotoxicity and that CAR-T cells are cultured *ex vivo*, where the functional status and the phenotype are not affected by the presence of CAR, unlike upon permanent CAR expression. However, *in vivo *experiments have revealed that the components of the tetracycline-responsive promoter system are immunogenic [68].

**Figure:**
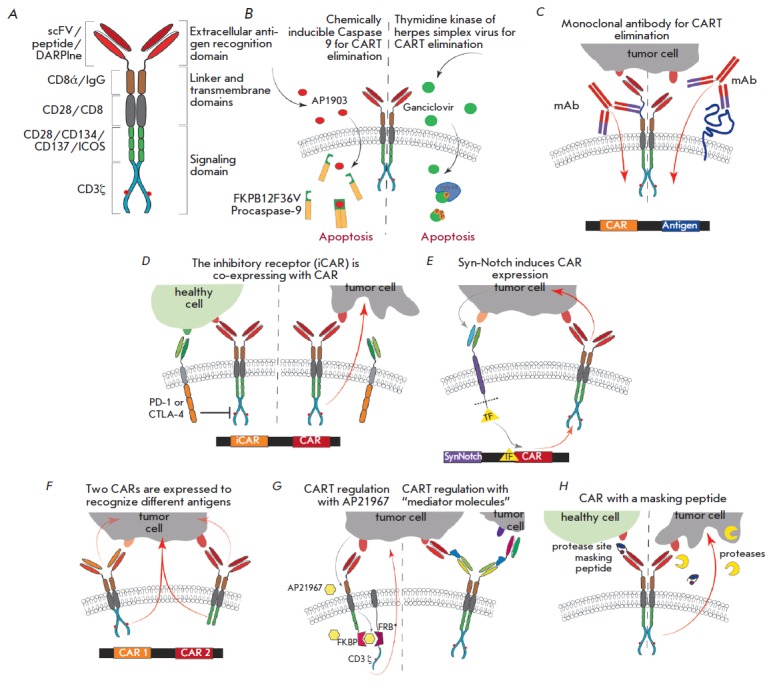
The methods used to regulate CAR T cells. *A *– the general structure of CAR. *B *– elimination of CAR by exogenous molecules. In the right-hand side of the Figure, HSV-TK phosphorylates ganciclovir to ganciclovir monophosphate, which is further sequentially converted to the di- and triphosphate forms by cellular kinases. Ganciclovir triphosphate is incorporated into DNA at the elongation and replication stages, resulting in cell death. In the left-hand side of the Figure, the truncated variant of caspase-9 and the FK506 fragment are dimerized in the presence of rimiducid (AP1903) and induce the apoptotic cascade. The antigen targeting monoclonal antibodies, which is capable of eliminating CAR T cells, is added to the surface of CAR T cells or to the linker domain of CAR. *D *– iCAR interacts with the antigen present on healthy cells and inhibits the CAR function via the intracellular domain of PD-1 or CTLA-4. This inhibition is reversible, which allows T cells to function when they subsequently encounter a tumor cell. *E *– after the additional receptor (synNotch) interacts with one tumor antigen, transcription factors (TFs) induce expression of CAR, which recognizes the second antigen and induces cytotoxicity. *F *– CAR T cells are sufficiently activated only when two CARs interact with two different tumor antigens. *G* – modular CARs. The left-hand side of the Figure shows that the activation ability of CARs is restored only upon dimerization of the protein binding FK 506 (FKBP) with the T2089L mutant of FKBP-rapamycin (FRB*) via the exogenously inserted rapamycin analogue (AP21967). In the right-hand side of the Figure: CAR is activated only through an exogenous “mediator molecule.” *H *– modification of the extracellular domain of CAR by a masking peptide, which is cleaved in the tumor microenvironment, thus allowing CAR to bind to its antigen.

## CONTROLLING THE ACTIVATION OF THE CHIMERIC ANTIGEN RECEPTOR

As already mentioned, chimeric antigen receptors consist of three key domains: the antigen recognition, the transmembrane, and the signaling domains. The direct relationship between antigen binding and receptor activation ensures the high efficiency of CAR T cells. In order to control the intensity of signal transmission from the antigen recognition domain to the signaling one, the receptor structure has been significantly modified by dividing it into two portions: the antigen-binding extracellular component and the intracellular component carrying the signaling domains. Both components carry the heterodimerization domains (FKBP and FRB*), which are hybridized in the presence of AP21967, a rapamycin analogue that is less immunosuppressive than rapamycin [70, 71]. Therefore, the immunoreactivity of therapeutic CAR T cells depends on the tumor antigen and the low-molecular-weight agent, whose concentration can be dosed (*Figure G*). An analysis of the therapeutic potential has demonstrated that AP21967-dependent CAR T cells and regular CAR T cells are equally effective, both *in vitro *and *in vivo *[65]. Meanwhile, this technique necessitates the design of novel classes of controller drugs optimized for clinical application in combination with therapeutically modified cells [65, 72-74].

## “MEDIATOR MOLECULES” HYBRIDIZING WITH THE EXTRACELLULAR CAR DOMAIN AND THE TUMOR ANTIGEN

It is possible to modulate both the intensity of signal transduction from the antigen recognition domain to the signaling one and the level of antigen recognition. The so-called “mediator molecules” (*Figure G*) show the greatest potential. These molecules are proteins or low-molecular-weight compounds with one end interacting with the tumor antigen and the other one interacting with CAR-modified T cells –– the so-called switchable (universal) CAR-T cells [75, 76]. The modularity of this approach allows one to broaden the range of antigens, while using the same CAR T cells. By adjusting the doses of “mediator molecules” one can regulate the intensity of the T-cell response and prevent the development of hypercytokinemia or the tumor lysis syndrome [77]. This strategy could be highly potent in polyclonal and recurrent tumors, when the T-cell response needs to be redirected [78, 79]. Either antibodies fused to a nonimmunogenic antigen targeted by CAR T cells or CARs targeting the Fc fragment of a therapeutic monoclonal antibody can be used as such “mediator molecules” [75-77, 80-84]. This approach has been implemented using recombinant anti-CD19 antibodies carrying the nonimmunogenic epitope of the GCN4 yeast transcription factor, which was in its turn targeted by the antigen recognition epitope of CAR T cells [77]. The same CAR T cells were successfully redirected using antibodies targeting CD20 modified by the GCN4 epitope [77]. The direct dependence between the phenotype of CAR T cells and concentration of mediator molecules was rather interesting: low doses of these molecules significantly increased the count of central memory T cells. Along with antibodies, modified natural polypeptides or their fragments carrying the hypervariable peptide segments responsible for molecular recognition can also be applied [85]. Well-known affinity pairs, such as the biotin–avidin pair, can also be used [76]. The same principle was employed to design fluorescein isothiocyanate (FITC)-conjugated antibodies targeting CD19 or FITC-conjugated folic acid. These “mediator molecules” are recognized by universal anti-FITC-CAR T cells [83, 86]. CD16-CAR T cells targeting the Fc domain of antibodies are currently being developed as universal CAR T cells. This will enable application of monoclonal antibodies in CAR T cell therapy [80-82].

Hence, switchable CAR T cells represent a promising new paradigm in cellular therapy which has the potential to enhance the safety and universality of CAR T cells. This approach will make production of CAR T cells simpler and reduce the cost of treatment. Being capable of redirecting therapy by changing “mediator molecules,” physicians could immediately adjust their treatment strategy. This method is especially relevant in preventing relapse after the development of mutations making the target tumor antigen disappear, as well as for effective therapy of tumors with heterogeneous expression of antigens [77, 79, 83]. Nevertheless, it remains disputable whether mediator molecules can be used in solid tumor therapy, since their tumor-penetrating ability is limited, which reduces the effectiveness of local activation and function of CAR T cells, while conventional CAR T cells can migrate into the tumor tissue [87, 88].

## MASKING THE ANTIGEN RECOGNITION DOMAIN OF THE CHIMERIC ANTIGEN RECEPTOR

The toxicity of CAR T therapy in dealing with solid tumors can be mitigated by modifying the antigen-binding domain of CAR with the masking peptide [89], which resides at the N-terminus of the chimeric antigen receptor, before the antigen-binding domain, and screens the recognition function of CAR (*Figure H*). A distinctive feature of some tumor types is that they contain specific proteases that hydrolyze the linker connecting the masking peptide and the antigen recognition domain of CAR. After the cleavage, CAR T cells can recognize the antigen presented on the tumor cell surface [89]. This approach enables use of the antigens presented on healthy cells for CAR-modified T cell therapy.

## APPLICATION OF MRNA TO MODIFY T CELLS

After they are administered to a patient, CAR T cells actively proliferate and differentiate into one of several T cell lineages. The new T cells also carry the CAR gene, which stimulates their activation. For most types of cancer, there is no need for the presence of therapeutic T cells during the entire life of a patient. Furthermore, it can cause additional complications and restoration of a patient’s immune status after therapy. Transfecting CAR-coding mRNA into T cells is one of the methods used to temporarily modify T cells with CARs [90]. This approach has been successfully used both *in vitro *and *in vivo *to study CD19- and mesothelin-specific CARs [90, 91]. Mesothelin-specific CARs have been subsequently successfully applied to treat pancreatic cancer [92, 93]. Electroporation of mRNA cells is carried out *in vitro *to avoid the potentially dangerous integration of the viral vector into a human’s genome [90, 91]. Unfortunately, a single infusion of CAR T cells is insufficient, which makes treatment more expensive and complex. However, multiple infusions of CAR T cells allow one to regulate the count of persisting cells and intensiveness of treatment [90] to avoid excessive cytokine release, the tumor lysis syndrome, and cytotoxicity with respect to healthy cells.

## CONCLUSIONS

The successful application of CAR-modified T cells *in vivo *and FDA approval of their use on patients with acute lymphoblast leukemia have made CAR T-cell therapy the most widely discussed and promising potential treatment for various types of cancer and even autoimmune diseases. However, a closer look and clinical trials have revealed that chimeric antigen receptors are not devoid of drawbacks and carry certain risks for patients. Therefore, it is safety and the possibility to control the therapy that matters most rather than its effectiveness. Many bioengineering techniques and approaches have been used to design next-generation CARs that are safer and can be controlled. Each of the reported approaches has its own advantages and drawbacks. However, thanks to the new approaches, cellular therapy can now be used at much earlier stages of cancer, thus significantly increasing the patient’s chances for a favorable outcome and reducing the risks of potential complications.
